# Fentanyl enhances immune cell response through TLR4/MD-2 complex

**DOI:** 10.3389/fphar.2024.1468644

**Published:** 2024-10-09

**Authors:** Chiara Chemello, Laura Facci, Emma Marcolin, Giovanni Eugenio Ramaschi, Massimo Barbierato, Pietro Giusti, Chiara Bolego, Morena Zusso

**Affiliations:** Department of Pharmaceutical and Pharmacological Sciences, University of Padua, Padua, Italy

**Keywords:** microglia, macrophages, fentanyl, inflammatory cytokines, TLR4/MD-2 complex

## Abstract

**Introduction:**

Opioids have been shown to induce neuroinflammation and immune cell activation, that might contribute to some of the opioid side effects, such as opioid-induced tolerance and paradoxical hyperalgesia. In this context, TLR4/MD-2 complex has been proposed as an off-target site for opioid action. This study was aimed at investigating the effect of fentanyl on lipopolysaccharide (LPS)-induced TLR4/MD-2 activation in rat primary microglia and human monocyte-derived macrophages (MDM).

**Materials and Methods:**

The effect of fentanyl was first explored by measuring the expression and release of different proinflammatory mediators in primary rat microglia and human MDM by real-time PCR and ELISA. Then, the involvement of TLR4/MD-2 signaling was investigated studying NF-κB activation in HEK293 cells stably transfected with human TLR4, MD-2, and CD14 genes (HEK-Blue hTLR4 cells) and in human MDM.

**Results:**

Fentanyl increased mRNA levels, as well as the LPS-induced secretion of proinflammatory mediators in primary microglia and MDM. Two inhibitors of TLR4/MD-2 signaling, namely the oxazoline derivative of *N*-palmitoylethanolamine (PEA-OXA) and CLI-095, blocked the production and release of proinflammatory cytokines by microglia stimulated with LPS and fentanyl, suggesting that TLR4/MD-2 could be the target of the proinflammatory activity of fentanyl. Finally, we showed that fentanyl in combination with LPS activated NF-κB signaling in human MDM and in HEK-Blue hTLR4 cells and this effect was blocked by inhibitors of TLR4/MD-2 complex.

**Discussion:**

These results provide new insight into the mechanism of the proinflammatory activity of fentanyl, which involves the activation of TLR4/MD-2 signaling. Our findings might facilitate the development of novel inhibitors of TLR4/MD-2 signaling to combine with opioid-based analgesics for effective and safe pain management.

## 1 Introduction

Chronic pain (*i.e.*, pain lasting ≥3 months) is a highly complex condition that represents a major public health problem, given its high prevalence, high rate of healthcare utilization, considerable society costs, and limited number of effective treatments. It negatively affects the quality of life of billions of people around the world and ∼20% of adult Europeans suffer from moderate to severe chronic pain (Raffaeli et al., 2021). Opioids, that act on the G protein-coupled receptors (GPCRs) µ, δ, and κ opioid receptors (or MOP, DOP, and KOP receptors, respectively), are among the most efficacious and widely used drugs for pain management. Their use for treating acute severe pain and chronic cancer pain is considered the mainstay approach (Casely and Laycock, 2022; Wang et al., 2023). However, the administration of opioids for the treatment of chronic non-cancer pain remains controversial, considering that long-term opioid use results in the development of unwanted side effects, including analgesic tolerance, addiction, and paradoxical hyperalgesia (Echeverria-Villalobos et al., 2023; Altawili et al., 2024).

The cellular and molecular mechanisms of such effects, as well as the complex neurobiology of pain, have been extensively studied, but are still not completely understood. Neurons and their circuits have long been considered the main target for the development of opioid tolerance (Williams et al., 2001). However, recent studies moved beyond the direct effect of opioids on neuronal functions and pointed to the role of both central and peripheral immune cells in some of the opioid adverse effects (Hutchinson et al., 2011; Grace et al., 2014a). In fact, opioids can induce neuroinflammation and activation of microglia, the primary immune cells of the CNS. Microglia activation initiates in response to a variety of neuronal injury, invading pathogens, and many other stressors. It is characterized by morphological changes, expression of molecular markers, and release of immunomodulatory molecules, such as cytokines, chemokines, and reactive oxygen and nitrogen intermediates (Woodburn et al., 2021). Neuroinflammation and microglia activation have been associated with the suppression of opioid analgesia and increased opioid tolerance (Cahill and Taylor, 2017; Han et al., 2022). In addition, in triple opioid receptor KO mice, opioids still induced microglia reactivity, tolerance, and hyperalgesia (Juni et al., 2007), suggesting the existence of an alternative non-opioid receptor that can mediate these effects. Neuroinflammation involves a coordinated response between microglia and monocyte-derived macrophages (MDM) infiltrating the CNS (Chen et al., 2023). However, the opioid effects on macrophages, that are the peripheral immune cell type with the closest functional relationship to microglia, have not been completely clarified (Ginhoux et al., 2010).

A possible site involved in opioid actions on central and peripheral immune system is the Toll-like receptor 4/myeloid differentiation factor 2 (TLR4/MD-2) complex (Machelska and Celik, 2020), which is abundantly expressed in innate immune cells, such as microglia and macrophages (O’Neill, 2008). TLR4/MD-2 can trigger two signaling pathways. The first one is activated in the plasma membrane by the adaptor proteins TIRAP and MyD88 and leads to the induction of proinflammatory molecules. The second begins in endosomes after endocytosis of the receptor and is activated by the adaptor proteins TRAM and TRIF and causes the induction of type I interferons (Kim et al., 2023).

A combination of experimental approaches has been used to study the off-target effect of opioids at TLR4. *In silico* docking simulations showed that morphine bound to the TLR4/MD-2 complex through the interaction with the LPS binding site on MD-2. Moreover, morphine non-stereoselectively activated TLR4/MD-2 signaling *in vitro*, which was blocked by a receptor antagonist (Wang X et al., 2012). Finally, *in vivo* pharmacological blockade or genetic removal of TLR4/MD-2 signaling potentiated acute opioid analgesia and attenuated the development of tolerance, hyperalgesia, and withdrawal behaviors (Bettoni et al., 2008; Hutchinson et al., 2010; Eidson and Murphy, 2013; Gabr et al., 2021). Collectively, these data suggest a role of TLR4/MD-2 in opioid actions. However, the pharmacological modulation of opioid-mediated TLR4 activation in immune cells and the intracellular pathways involved remain to be fully characterized.

Here we provide evidence that fentanyl, a potent synthetic μ-stimulating opioid approved for use as an analgesic and anesthetic with a high rate of abuse potential, increased LPS-induced inflammation in rodent microglia and human MDM. We also showed that inhibitors of TLR4/MD-2, previously identified by others and us (Ii et al., 2006; Facci et al., 2023) reduced fentanyl-induced microglia activation and TLR4/MD-2 signaling. These results provide new insight into the mechanism of the proinflammatory activity of fentanyl, which involves the activation of TLR4/MD-2 signaling. Our findings might facilitate the development of new molecules active in modulating opioid-mediated TLR4 activation, for future pharmacological interventions to improve the analgesic effect of opioids and decrease their unwanted side effects.

## 2 Materials and methods

### 2.1 Materials

Fentanyl citrate (#F3886, Lot SLCL4520), naloxone hydrochloride dihydrate (#N7758, Lot SLBF5550V), all chemicals, tissue culture media, and antibiotics, unless otherwise specified, were from Merck (Milan, Italy). Fetal bovine serum (FBS) was obtained from Life Technologies (San Giuliano Milanese, Italy). Macrophage colony stimulating factor (MCSF) was from ImmunoTools (Friesoythe, Germany). LPS (Ultrapure LPS-EB from *Escherichia Coli*, 0111:B4 strain; # tlrl-3pelps, Lot 5970-45-01) was purchased from InvivoGen (InvivoGen Europe, Toulouse, France); this ultrapure preparation does not contain other bacterial components (Supplementary Figure S1) and only activates TLR4 (Hirschfeld et al., 2000). Ethyl (6*R*)-6-[*N*-(2-chloro-4-fluorophenyl)sulfamoyl]cyclohex-1-ene-1-carboxylate (CLI-095 also known as TAK-242; # tlrl-cli95, Lot 6145-42-01), an inhibitor of TLR4 signaling, was from InvivoGen. *N*-Palmitoylethanolamine-oxazoline (PEA-OXA), kindly provided by Epitech Group (Saccolongo, Italy), was dissolved in dimethyl sulfoxide (DMSO)/ethanol (70/30 v/v) and used with a final concentration of 0.07% DMSO and 0.03% ethanol. All water-soluble reagents were dissolved in endotoxin-free water (InvivoGen).

### 2.2 Cell cultures

Sprague-Dawley rats (CD strain) were maintained under controlled temperature and humidity, with free access to water and food on a 12-h light/dark cycle (lights on at 7:00 a.m.). Animal-related procedures followed Italian Ministry of Health guidelines (D.L. 26/2014) for the care and use of laboratory animals and were approved by the Institutional Review Board for Animal Research (Organismo Preposto al Benessere Animale, OPBA) of the University of Padua and by the Italian Ministry of Health. Primary microglial cells were isolated from mixed glial cell cultures prepared from cerebral cortices of PN1 rat pups, as previously described (Facci et al., 2018). In brief, when mixed glial cultures reached confluence (typically 7 days after isolation), microglia were recovered by shaking the flasks on an orbital shaker (200 rpm for 1 h at 37°C), re-suspended in high-glucose Dulbecco’s modified eagle medium (DMEM) supplemented with 2 mM L-glutamine, 10% heat-inactivated FBS, 100 units/mL penicillin, 100 μg/mL streptomycin, and 50 μg/mL gentamicin (growth medium), transferred to Sterilin plastic Petri dishes, and incubated for 45 min at 37°C (5% CO_2_, 95% air) to allow adhesion of microglia. The adherent microglial cells (>99% pure, as determined by flow cytometry using cell type-specific antibodies (Marinelli et al., 2015)) were detached by mechanically scraping into growth medium and re-plated in this same medium, on poly-L-lysine-coated plastic wells at a density of 1.50 × 10^5^ cells/cm^2^.

Human MDM were obtained from buffy coats of male and female donors provided by the University of Padova Medical Center Transfusion Unit, following institutional standard operating procedures as previously described (Tedesco et al., 2018a). Briefly, monocytes were isolated by Ficoll-Paque followed by a second centrifugation using high-density hyperosmotic Percoll. The cells were seeded at a density of 2 × 10^5^ cells/cm^2^. After 24 h, non-adherent cells were removed, and the adherent monocytes were maintained in RPMI-1640 with 2 mM L-glutamine, 10% heat-inactivated FBS, 100 units/mL penicillin, 100 μg/mL streptomycin, and 20 nM MCSF. To obtain MDM, monocytes were cultured for 7 days with medium changes every 3 days.

HEK-Blue™ hTLR4 cells, obtained by co-transfection of the human TLR4, MD-2 and cluster of differentiation 14 (CD14) co-receptor genes, and an NF-κB-inducible secreted embryonic alkaline phosphatase (SEAP) reporter gene into human embryonic kidney 293 (HEK293) cells, were purchased from InvivoGen. Cells were cultured in high-glucose DMEM supplemented with 2 mM L-glutamine, 10% FBS, 100 units/mL penicillin, 100 μg/mL streptomycin, 100 μg/mL Normocin™ (InvivoGen), and 1× HEK-Blue™ selection (InvivoGen) (selection medium), according to the supplier’s instructions. When the cells reached the confluency of ∼80%, they were subcultured and plated at a density of 0.1 × 10^6^ cells/mL.

Cells were maintained at 37°C in a humidified atmosphere containing 5% CO_2_/95% air.

### 2.3 Cytokine determination

At the end of treatments, culture media from rat microglia and human MDM were collected and interleukin (IL)-1β and tumor necrosis factor (TNF)-α assayed using commercially available ELISA kits for rat and human samples (Antigenix America, Huntington Station, NY, United States), according to the manufacturer’s instructions. The absorbance of each sample was detected at 450 nm with a microplate reader. Cytokine concentrations (pg/mL) in the medium were determined by reference to standard curves obtained with known amounts of IL-1β or TNF-α (Bisceglia et al., 2019).

### 2.4 Nitric oxide assay

Primary microglia were exposed to 10 μM fentanyl in the absence or presence of 10 ng/mL Ultra-Pure LPS-EB for 24 h. Thereafter, the production of nitric oxide (NO) was determined by the indirect measurement of its stable oxidized products, nitrite and nitrate, using the Griess reaction. Fifty µL of the cell culture medium and an equal volume of the Griess reagent were mixed, incubated at room temperature for 15 min, and the absorbance at 540 nm was measured in a microplate reader. The nitrite concentration in the supernatant was quantified using a standard curve of sodium nitrite.

### 2.5 Cell reporter assay

HEK-Blue™ hTLR4 cells were plated at a density of 0.4 × 10^5^ cells per well using 200 μL/well (96-well-plate) of selection medium and allowed to adhere overnight. Thereafter, selection medium was replaced with serum-free medium and cells were subjected to different treatments for 24 h. Supernatants were collected and SEAP released in the culture medium was quantified using the QUANTI-Blue™ Solution (InvivoGen). Briefly, 20 μL of supernatants were incubated with 180 μL of the QUANTI-Blue™ Solution in a 96-well plate for 15 min. SEAP activity, as an indicator of TLR4/MD-2 complex activation, was assessed by reading the optical density at 630 nm (OD_630_) with a microplate reader.

### 2.6 Real-time polymerase chain reaction (real-time PCR)

Total RNA was extracted using and RNA extraction mini kit (Qiagen, Milan, Italy). RNA integrity and quantity were determined by RNA 6000 Nano assay (A_260/280_ ratio > 1.8; Thermo Scientific, Milan, Italy). First strand cDNA was synthesized from 1 μg total RNA using Superscript IV reverse transcriptase or Maxima first strand cDNA synthesis kit (Thermo Fischer Scientific), according to the manufacturer’s instructions. Real-time PCR reaction was performed using SYBR Green JumpStart Taq ReadyMix in a AriaMX thermal cycler (Agilent Technologies, Santa Clara, CA, United States) or in a QuantStudio 3 Real-Time PCR (Applied Biosystems, Thermo Fisher Scientific). Thermal cycling conditions have been optimized for the two thermal cyclers: initial denaturation at 94°C for 5 min, followed by 40 cycles of 94°C for 30 s, 60°C for 30 s, and 72°C for 30 s for the AriaMX thermal cycler; 40 cycles of denaturation at 95°C for 15 s, annealing at 60°C for 30 s, and extension at 72°C for 30 s, for QuantStudio 3 Real-Time PCR. Rat and human primer sequences are listed in Table 1, 2, respectively. Data were analyzed using the comparative 2^−ΔΔCT^ method and normalized to β-actin or GAPDH mRNA level. Dissociation curves were generated for each primer pair, showing single product amplification. Results are shown as fold changes compared to control value.

**TABLE 1:** Rat primer sequences used in this study.

Gene target	Primer name	Sequence (5′-3′)
β-actin	β-actin For	GAT​CAG​CAA​GCA​GGA​GTA​CGA​TGA
	β-actin Rev	GGT​GTA​AAA​CGC​AGC​TCA​GTA​ACA
IL-1β	IL-1β For	CGT​CCT​CTG​TGA​CTC​GTG​GG
	IL-1β Rev	ATG​GGT​CAG​ACA​GCA​CGA​GG
iNOS	iNOS For	GGG​AAC​ACC​TGG​GGA​TTT​TC
	iNOS Rev	CAC​AGT​TTG​GTC​TGG​CGA​AG
TNF-α	TNF-α For	GCA​GGT​TCC​GTC​CCT​CTC​AT
	TNF-α Rev	TGC​CAG​TTC​CAC​ATC​TCG​GA
DOP	OPRD1 For	GGA​CGC​TGG​TGG​ACA​TCA​AT
	OPRD1 Rev	GTA​GAG​AAC​CGG​GTT​GAG​GC
KOP	OPRK1 For	TGC​GTG​GCC​TTT​CTG​TGT​AA
	OPRK1 Rev	TGT​TAA​CGC​GAA​GGT​TGG​GT
MOP	OPRM1 For	ATT​GCA​CCC​TCA​CGT​TCT​CC
	OPRM1 Rev	CCG​GCA​TGA​TGA​AAG​CGA​AG
NOP	OPRL1 For	GTA​TTG​CAG​TGG​GGA​GCA​TTA
	OPRL1 Rev	CCT​CGG​GTA​ATC​TGA​CTG​CAT

**TABLE 2:** Human primer sequences used in this study.

Gene target	Primer name	Sequence (5′-3′)
GAPDH	β-actin For	CAC​CAT​CTT​CCA​GGA​GCG​AG
	β-actin Rev	CCT​TCT​CCA​TGG​TGG​TGA​AGA​C
IL-1β	IL-1β For	GGG​CCT​CAA​GGA​AAA​GAA​TC
	IL-1β Rev	TTC​TGC​TTG​AGA​GGT​GCT​GA
TNF-α	TNF-α For	TCC​TTC​AGA​CAC​CCT​CAA​CC
	TNF-α Rev	AGG​CCC​CAG​TTT​GAA​TTC​TT
DOP	OPRD1 For	GCC​TCG​GAC​GCC​TAC​CCT​A
	OPRD1 Rev	GAG​TAG​AGC​GCG​GTG​ATG​G
KOP	OPRK1 For	CCA​TCC​CGG​TCA​TCA​TCA​CG
	OPRK1 Rev	ATC​ACG​AAC​ATG​ACC​AGC​GA
MOP	OPRM1 For	TGC​CCT​TCC​AGA​GTG​TGA​AT
	OPRM1 Rev	CAG​AGG​GTG​AAT​ATG​CTG​GTG​AA
NOP	OPRL1 For	ACA​TCT​TTA​ACC​TGG​CCC​TGG​C
	OPRL1 Rev	CAT​TCC​CAA​ACG​GCC​AGA​AGC

### 2.7 SDS PAGE and western blotting

At the end of treatments, MDM were lysed with lysis buffer (PBS supplemented with 1.2% Triton X-100, 1× Roche Complete™ inhibitor cocktail, 2.5 mM NaF, 2 mM sodium pyrophosphate, 4 mM Na orthovanadate, 1 mM PMSF). After centrifugation at 10,000 × g for 15 min, supernatants were collected and the protein concentration was determined by the BCA assay (Euroclone, Milan, Italy). Total protein lysates (30–40 μg) were separated on 10% SDS-PAGE and transferred onto PVDF membranes (Hybond-P, Amersham, Little Chalfont, United Kingdom). Membranes were then blocked with 5% bovine serum albumin followed by incubation overnight at 4°C with primary antibodies against IκBα (rabbit polyclonal, 1:1,000, #9242, Lot 12, Cell Signaling Technology, Danvers, MA, United States), phospho–IκBα (mouse monoclonal, 1:1,000, #9246, Lot 23, Cell Signaling Technology), and GAPDH (rabbit polyclonal 1:10,000, ab181602, Lot GR3219790-4, Abcam, Cambridge, United Kingdom). After washing, membranes were incubated with goat anti-rabbit or horse anti-mouse secondary HRP-conjugated antibodies (Vector Laboratories, Burlingame, CA, United States) at 1:5000 dilution. Bands were detected by chemiluminescence using the Clarity Western ECL substrate (Bio-Rad, Hercules, California, United States). Images were acquired with C400 Azure Imaging System (Azure Biosystem, Dublin, CA, United States). Densitometric analysis of the bands was performed with ImageJ version 1.47. Data are expressed as arbitrary units of OD and represent mean values of 5 independent experiments.

### 2.8 Statistical analysis

All data represent the results of at least three independent experiments performed in triplicate. Results are expressed as mean ± standard error of the mean (SEM). GraphPad Prism Software, version 8.4 (San Diego, CA, United States) was used for plotting of the data and statistical analysis. Data were analyzed by one-way ANOVA followed by *post hoc* tests, as detailed in the figure legends. Non-linear regression was used to plot and analyze concentration–response curves and to obtain EC_50_ and E_max_ values. Differences were considered statistically significant when *p* < 0.05 or as evidenced by non-overlapping 95% confidence intervals (CI).

## 3 Results

### 3.1 Effect of fentanyl on LPS-induced microglia inflammatory response

**FIGURE 1:**
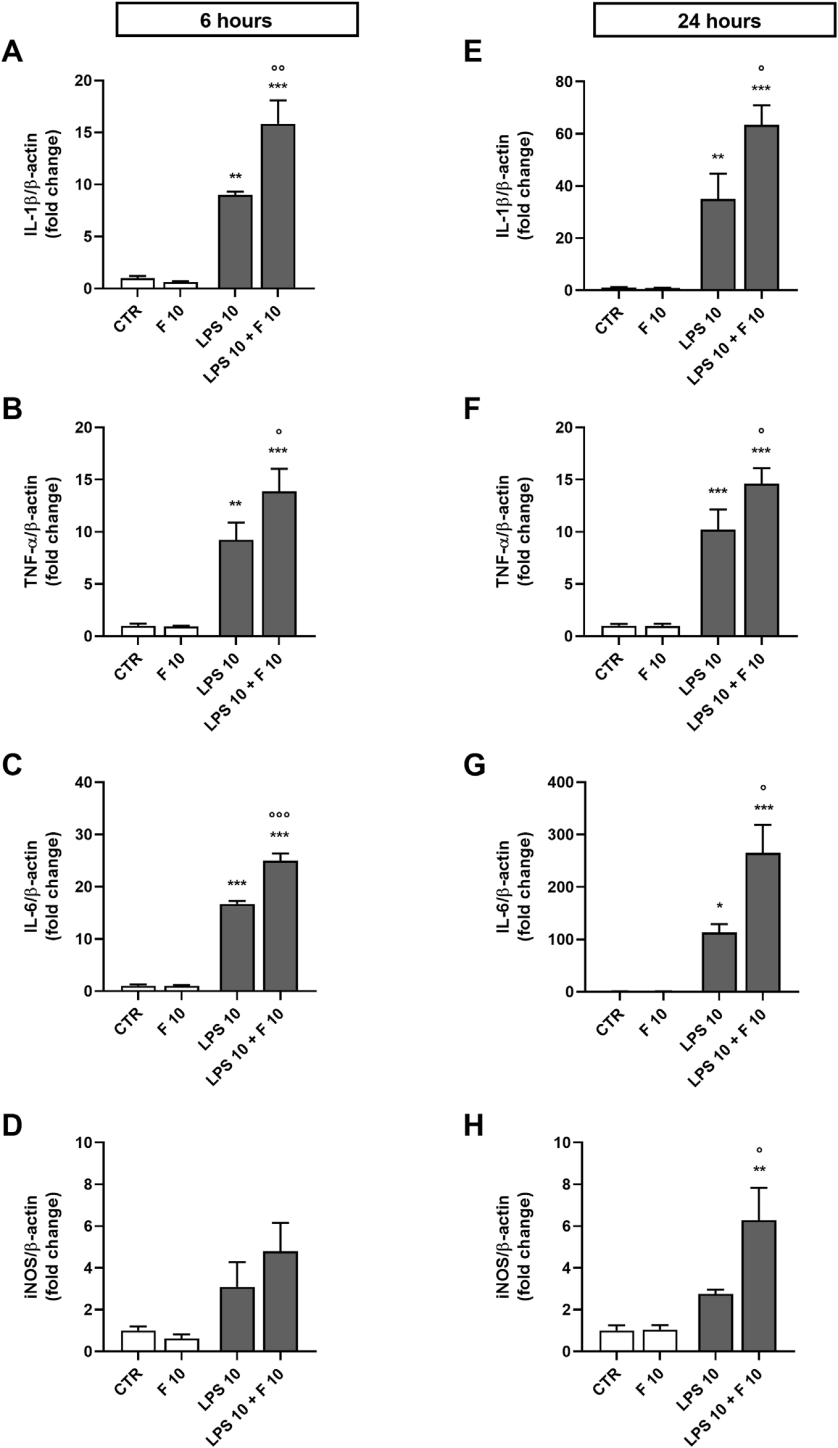
Effect of fentanyl on proinflammatory gene expression in primary cortical microglia. Microglia were cultured in 10% serum-containing medium, which was replaced with serum-free medium before treatment with 10 μM fentanyl (F) for **(A-D)** 6 h or **(E-H)** 24 h in the absence (white bars) or presence (gray bars) of 10 ng/mL LPS. Gene expression was quantified by real-time PCR. Data are presented as means ± SEM (n = 3) and analyzed by one-way ANOVA followed by Holm-Sidak’s multiple comparison test. *p < 0.05, **p < 0.01, and ***p < 0.001 compared to control cells (CTR); °p < 0.05, °°°p < 0.01, °°°p < 0.001 vs*.* LPS stimulation.

**FIGURE 2:**
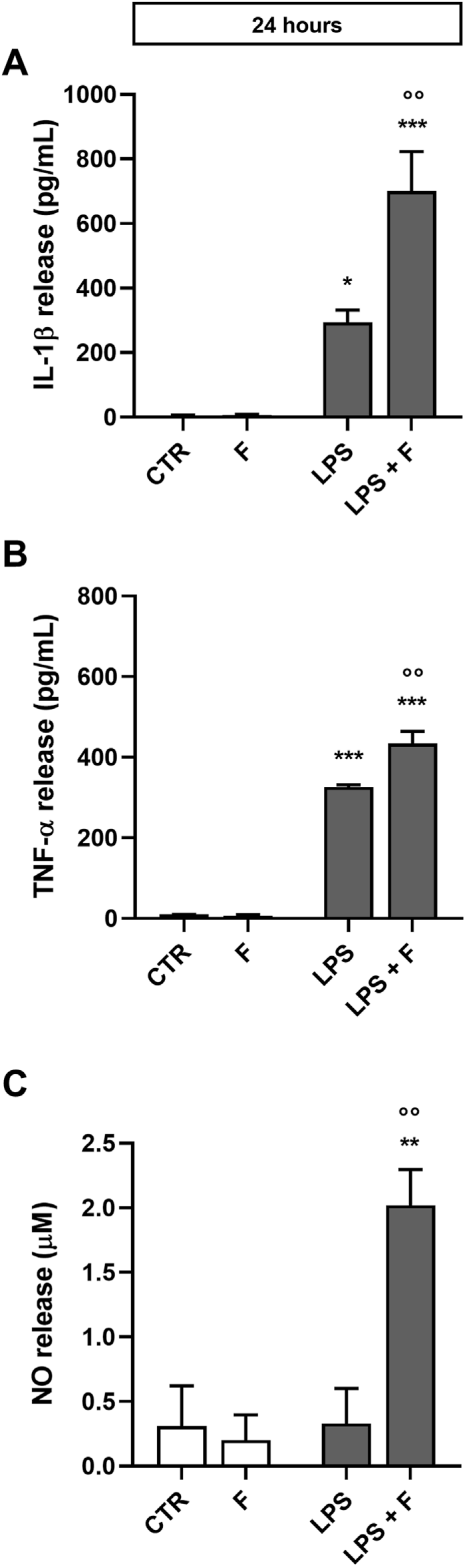
Effect of fentanyl on proinflammatory mediator release from primary cortical microglia. Microglia were cultured in 10% serum-containing medium, which was replaced with serum-free medium before treatment with 10 μM fentanyl (F) for 24 h in the absence (white bars) or presence (gray bars) of 10 ng/mL LPS. Supernatants were collected and analyzed for **(A)** IL-1β, **(B)** TNF-α, and **(C)** NO release. Results are shown as means ± SEM (n = 3) and analyzed by one-way ANOVA followed by Holm-Sidak’s multiple comparison test. *p < 0.05, **p < 0.01, and ***p < 0.001 compared to control cells (CTR); °p < 0.01 vs*.* LPS stimulation.

**FIGURE 3:**
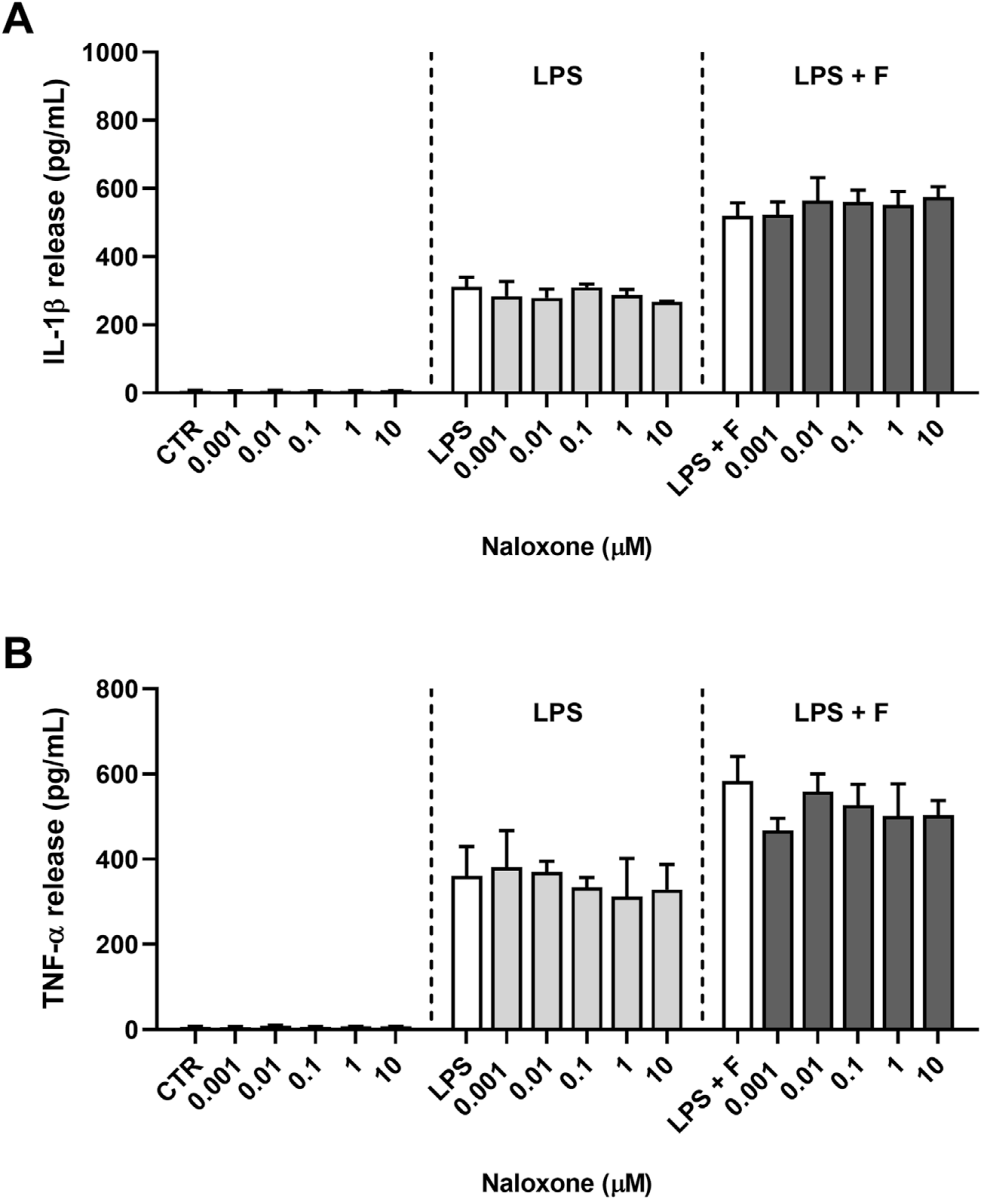
Effect of naloxone on proinflammatory cytokine release from primary cortical microglia. Microglia were cultured in 10% serum-containing medium, which was replaced with serum-free medium before treatments for 24 h. Supernatants were collected and analyzed for **(A)** IL-1β and **(B)** TNF-α release. *Left panels*: cells were treated with naloxone alone (0.001–10 μM). *Middle panels*: cells were pre-treated for 30 min with naloxone (0.001–10 μM) and the stimulated with 10 ng/mL LPS. *Right panels:* cells were pre-treated for 30 min with naloxone (0.001–10 μM) and then stimulated with 10 ng/mL LPS and fentanyl (F, 10 μM). White bars show the effect of control treatment for each panel. Results are shown as means ± SEM (n = 3) and analyzed by one-way ANOVA.

Initial studies were done to explore the effect of fentanyl on the neuroinflammatory response of microglia. For this purpose, we investigated the drug effect on the expression of some inflammatory signal messengers in microglia cultures when stimulated with the TLR4 ligand LPS. For these studies we used a concentration of LPS that activated microglia without eliciting the maximal response (*i.e*., 10 ng/mL; see Supplementary Figure S2 for a concentration-response curve of LPS). Fentanyl tested alone at the non-cytotoxic concentration of 10 μM (Supplementary Figure S3) had no effect on the low basal levels of IL-1β, TNF-α, IL-6, and inducible nitric oxide synthase (iNOS) (white bars in Figure 1). Differently, co-treatment with 10 ng/mL LPS resulted in a significant increase of microglia activation. In particular, fentanyl further increased the mRNA levels of IL-1β, TNF-α, and IL-6 produced by LPS alone after a 6- and 24-h treatment (Figures 1A–C, E–G, respectively). In addition, the expression of iNOS increased after treatment with fentanyl and LPS for 24 h (Figure 1H), while no effect was observed after 6 h (Figure 1D). Changes in gene expression were accompanied by the induction of cytokine and NO release in the culture medium after a stimulation for 24 h (Figure 2). In addition, it should be noted that the opioid receptor antagonist naloxone did not affect microglia activation. Specifically, Figure 3 shows that naloxone (0.001–10 μM) did not modify microglia activation induced by LPS alone (middle panels) and by LPS plus fentanyl (right panels), thus supporting the hypothesis that fentanyl acts through an opioid receptor-independent pathway.

### 3.2 Effect of TLR4/MD-2 inhibition on the proinflammatory activity of fentanyl in microglia

**FIGURE 4:**
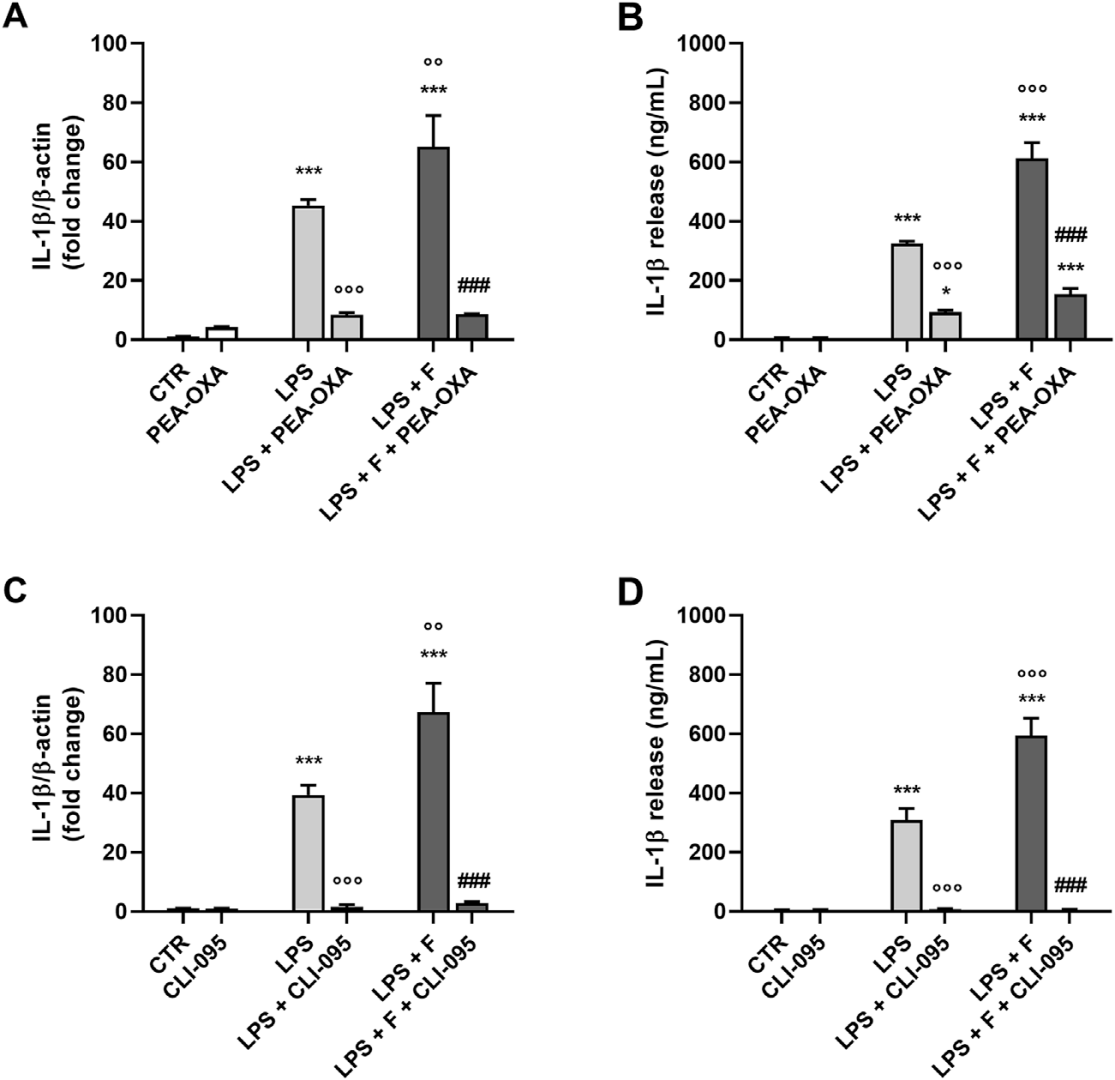
Effect of TLR4/MD-2 inhibition on the proinflammatory activity of fentanyl. Microglia were cultured in 10% serum-containing medium, which was replaced with serum-free medium before treatment with fentanyl (F, 10 μM) and LPS (10 ng/mL), in the presence of **(A, B)** PEA-OXA (30 μM) or **(C, D)** CLI-095 (0.5 μg/mL) for 24 h **(A, C)** IL-1β mRNA levels were quantified by real-time PCR. **(B, D)** Supernatant were collected and analyzed for IL-1β content. Data are means ± SEM (n = 3). ***p ˂ 0.001 vs*.* control cells (CTR); °°p < 0.01 and °°°p < 0.001 vs*.* LPS; ^###^p < 0.001 vs*.* LPS + fentanyl. One-way ANOVA followed by Holm-Sidak’s test.

To determine the target of fentanyl at receptor level, we explored whether TLR4/MD-2 complex could be required for the proinflammatory effect of the opioid drug. First, the receptor signaling was blocked using PEA-OXA, a TLR4/MD-2 inhibitor that can stably accommodate in the LPS recognition site in the MD-2 structure (Facci et al., 2023). PEA-OXA at 30 μM concentration reduced the production and release of IL-1β from microglia stimulated with LPS alone (used as positive control; Figures 4A, B, light gray bars) and by the co-treatment with LPS plus fentanyl (Figures 4A, B, dark gray bars), suggesting that TLR4/MD-2 complex could be the target for proinflammatory effects of fentanyl.

Next, TLR4/MD-2 signaling was blocked by CLI-095, a cyclohexene derivative that suppresses specifically TLR4 signaling by binding to the receptor intracellular domain (Ii et al., 2006; Kawamoto et al., 2008; Takashima et al., 2009). CLI-095 (0.5 μg/mL) completely inhibited IL-1β release and its mRNA expression in microglia stimulated with LPS and fentanyl (Figures 4C, D, dark gray bars), further suggesting that the proinflammatory effect of fentanyl is dependent on TLR4/MD-2 activation.

### 3.3 Effect of fentanyl on TLR4/MD-2 activation

**FIGURE 5:**
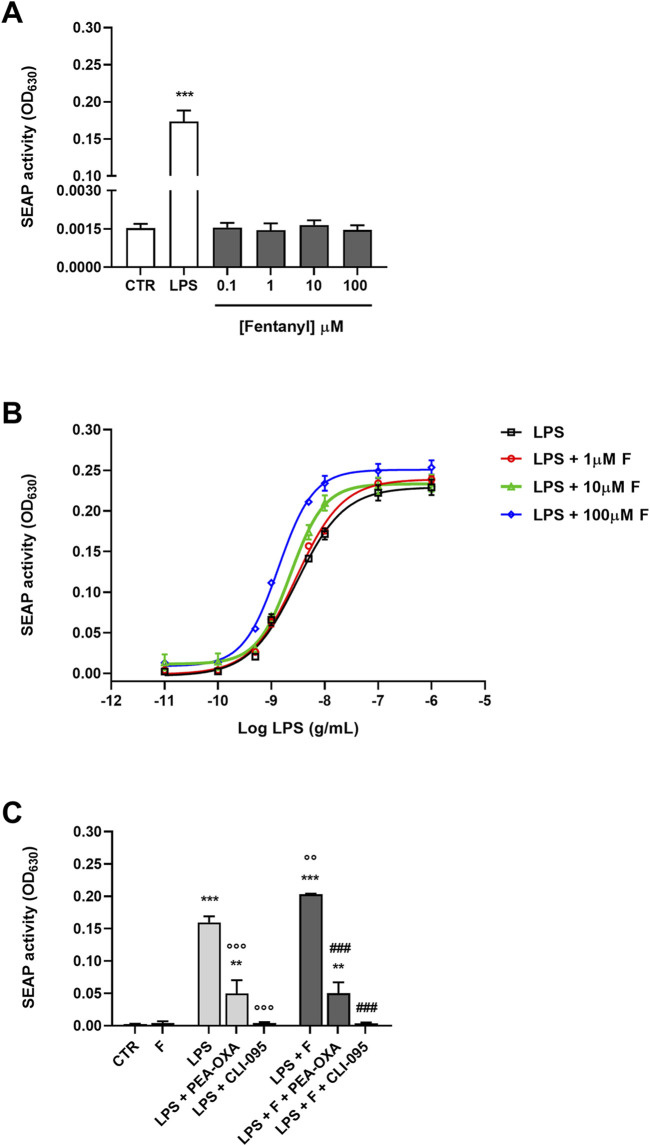
Effect of fentanyl on TLR4/MD-2 activation. **(A)** HEK-Blue hTLR4 cells were incubated with fentanyl (0.1–100 μM) and the amount of SEAP released into the culture medium was quantified after 24 h **(B)** HEK-Blue hTLR4 cells were treated with LPS alone (from 10^−11^ to 10^−6^ g/mL) or co-treated with fentanyl (F) at increasing concentrations (1–100 µM) and the amount of SEAP released into the culture medium was quantified after 24 h. EC_50_ and E_max_ values are given in Table 4. **(C)** HEK-Blue hTLR4 cells were co-treated with 100 μM fentanyl and 5 ng/mL LPS, in the absence or presence of 30 μM PEA-OXA or 0.5 μg/mL CLI-095 for 24 h. Data are shown as OD_630_ and are means ± SEM (n = 4). **p < 0.01 and ***p < 0.001 vs*.* control cells (CTR); °°p < 0.01 and °°°p < 0.001 vs*.* LPS; ^###^p < 0.001 vs*.* LPS + fentanyl. One-way ANOVA followed by Holm-Sidak’s test.

Since we found that opioid receptor mRNAs were expressed in primary microglia (Table 3), the role of TLR4/MD-2 was further tested using HEK-Blue hTLR4 cells, that do not express any endogenous opioid receptors (Gharagozlou et al., 2002; Stevens et al., 2013). These cells are co-transfected with the hTLR4, hMD-2, and hCD14 receptor genes along with a SEAP reporter gene. When TLR4/MD-2 is stimulated, NF-κB is activated through an intracellular pathway, leading to the release of SEAP that can be detected in the culture medium with a colorimetric assay. Initially, HEK-Blue hTLR4 cells were treated with increasing concentrations of fentanyl alone (0.1–100 μM). LPS was used as positive controls for hTLR4/MD-2 activation. Results in Figure 5A show that fentanyl did not activate TLR4/MD-2 signaling. Then, HEK-Blue hTLR4 cells were treated with increasing concentrations of LPS (10^−11^–10^−6^ g/mL) that produced a concentration–dependent increase in receptor activation with an EC_50_ of 3.00 ng/mL (95% CI ranging from 2.37 to 3.77). Concurrent treatment with fentanyl caused a leftward shift of the LPS curve, with fentanyl at 100 μM concentration producing a significant lower EC_50_ value of 1.35 ng/mL (95% CI ranging from 1.16 to 1.60) (Figure 5B), without affecting E_max_ of all the concentration–response curves (Table 4).

**TABLE 3:** Opioid receptor expression in rat cortical microglia.

Receptor	Rat cortical microglia
MOP	1.00 ± 0.16
DOP	0.18 ± 0.01
KOP	0.68 ± 0.09
NOP	0.36 ± 0.09

Real-time PCR was used to measure the mRNA, expression of opioid receptors. Data were normalized to β-actin mRNA level and results are shown relative to MOP receptor mRNA levels set to 1. Data are means ± SEM of three independent experiments.

**TABLE 4:** Pharmacological parameters of TLR4 stimulation by LPS alone and with different concentrations of fentanyl (F) co-treatment.

Treatment	EC_50_ ^a^	95% CI	E_max_	95% CI
LPS alone	3.00	2.37–3.77	0.23	0.21–0.24
LPS + F (1 µM)	2.96	2.57–3.41	0.24	0.23–0.25
LPS + F (10 µM)	2.29	1.77–2.95	0.23	0.22–0.25
LPS + F (100 µM)	1.35^b^	1.16–1.60	0.25	0.23–0.26

^a^ Effective concentration for 50% TLR4 signaling is given in ng/mL.

^b^ Denotes significantly different from concentration-response curve of LPS alone.

Finally, addition of PEA-OXA and CLI-095 to the fentanyl plus LPS treatment resulted in a significant inhibition of TLR4/MD-2 signaling (Figure 5C).

### 3.4 Effect of fentanyl on LPS-induced macrophage inflammatory response

**FIGURE 6:**
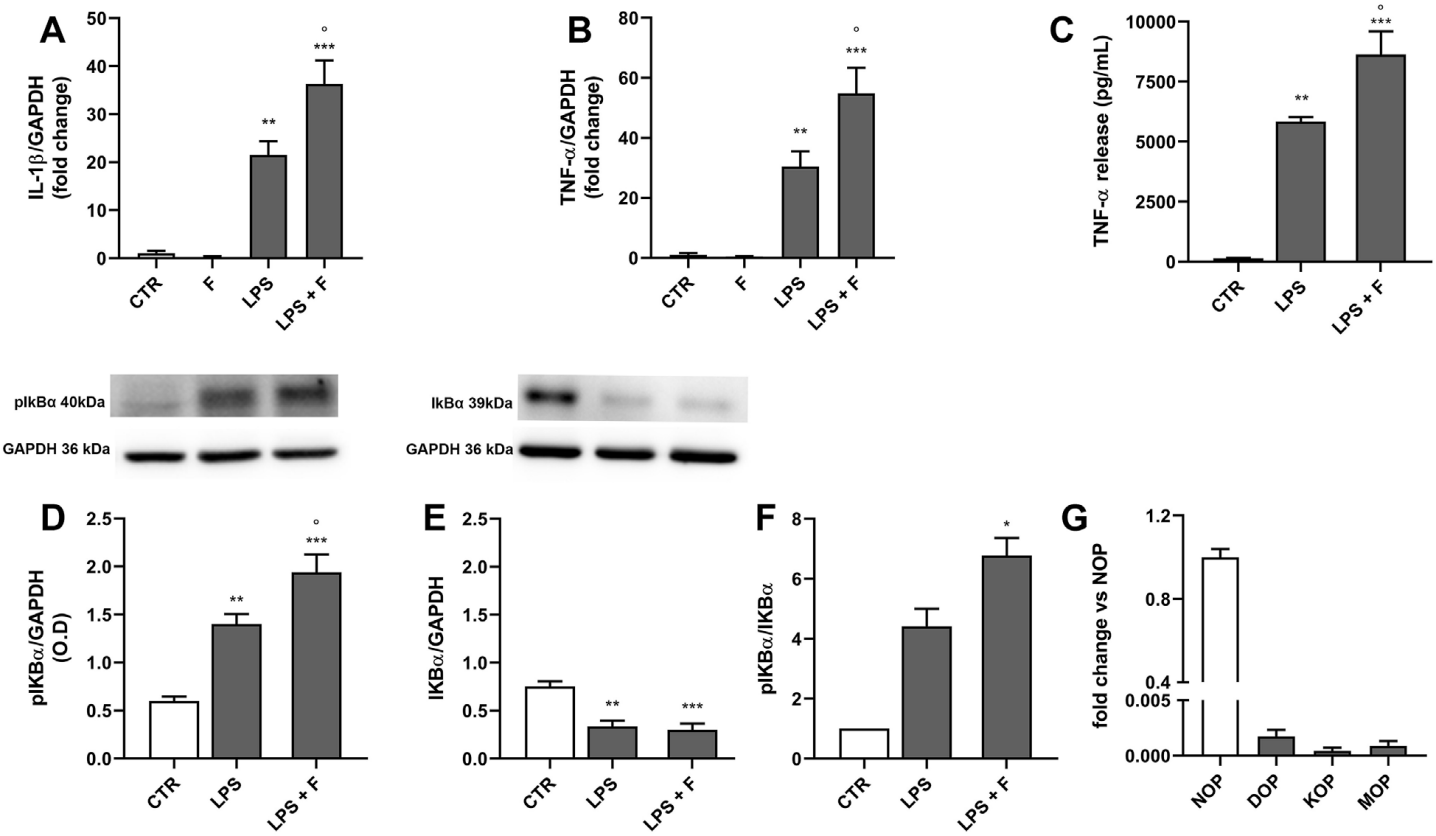
Effect of fentanyl on LPS-induced MDM inflammatory response. Human MDM were cultured in 10% serum-containing medium, which was replaced with serum-free medium before treatment with 10 μM fentanyl (F) in the absence (white bars) or presence (gray bars) of 100 ng/mL LPS. **(A, B)** IL-1β and TNF-α mRNA levels were quantified by real-time PCR after 3-h stimulation. **(C)** TNF-α release was measured by ELISA in MDM supernatants collected after 24-h stimulation. **(D–F)** pIkB and IkB protein levels were analyzed by Western blot after 90-min stimulation. GAPDH was used as loading control. **(G)** The opioid receptor gene expression was quantified by real-time PCR and shown relative to NOP receptor mRNA levels set to 1. Data are presented as means ± SEM of 3–5 independent experiments and analyzed by one-way ANOVA followed by Sidak’s multiple comparison test. **p < 0.01 and ***p < 0.001 compared to control (CTR); °p < 0.05 vs*.* LPS stimulation. O.D., optical density in arbitrary units.

To further explore TLR4-mediated effects of fentanyl on immune cells, we tested MDM from human blood. We first evaluated the expression of IL-1β and TNF-α in cells treated with 10 µM fentanyl in the presence or absence of LPS for 3 h. As shown in Figures 6A, B, fentanyl alone did not affect IL-1β or TNF-α mRNA levels. However, similarly to what observed in rat microglia, fentanyl significantly increased LPS-induced expression of both cytokines. Consistently, 24-h treatment with fentanyl resulted in a significantly increased release of TNF-α into the medium with respect to LPS alone (Figure 6C).

Since the expression of several inflammatory cytokines *via* TLR4 signaling involves NF-κB activation and nuclear translocation, we next explored the effect of fentanyl combined with LPS on IκB phosphorylation as a pathway activation readout (Tedesco et al., 2018b; Mussbacher et al., 2023). Figures 6D, E shows that the phospho IκB levels were significantly higher in macrophages treated for 90 min with LPS plus fentanyl compared to LPS alone, whereas IκB levels decreased. Accordingly, phopsho IκB/IκB ratio was higher in MDM challenged with LPS plus fentanyl compared to LPS alone (Figure 6F), suggesting that fentanyl and LPS activate a common proinflammatory signaling.

We also found that human macrophages express barely detectable mRNA levels of MOP, DOP, and KOP receptors, while expressing the nociception/orfanin FQ opioid peptide (NOP) receptor (Figure 6G). Overall, these data support the hypothesis that fentanyl amplifies LPS effect in MDM by interacting with TLR4/MD-2.

## 4 Discussion

Opioids are the most powerful analgesics available for pain treatment. However, when used chronically, they can cause severe side effects and clinical problems, such as sedation, nausea, vomiting, constipation, addiction, tolerance, and respiratory depression. Some of these effects have been correlated with the ability of opioid ligands to affect peripheral and central immune cell functions. Once activated, these cells can contribute to an exacerbation of proinflammatory and pro-nociceptive processes and promote, over the long-term, opioid-induced hyperalgesia and tolerance (Varrassi et al., 2018; Echeverria-Villalobos et al., 2023). Despite the traditional view of opioids being immunosuppressive, recent studies indicate variable effects of endogenous and exogenous opioids on immune functions, mainly based on exposure time (Plein and Rittner, 2018; Wen et al., 2022; Sun et al., 2023). Of particular importance in the opioid modulation of immune cell activities is TLR4/MD-2 complex. In the CNS, opioid agonists can bind to this receptor, activate its intracellular signaling, and induce a central immune response (Grace et al., 2014b; Grace et al., 2015). Similarly, some studies pointed to a non-GPCR opioid site of action also in peripheral immune cells (Roy et al., 1998).

Here, we first examined the effect of fentanyl on LPS-stimulated primary rat microglia, the key cells involved in immune responses in the CNS. We showed that fentanyl potentiated LPS-induced microglia activation, which was evident by elevated mRNA expression levels and release of proinflammatory mediators. Numerous studies have already shown that opioid agonists, especially morphine and fentanyl, two of the most clinically relevant opioids, lead to microglia activation. For instance, the expression and production of proinflammatory cytokines from primary mouse microglia and mouse BV-2 microglial cell line markedly increased by morphine treatment (Wang Q et al., 2012; Liang et al., 2016; Pan et al., 2016; Yang et al., 2021). However, only a few studies have examined the effect of opioids on LPS-stimulated microglia and most of them have shown that opioids differentially modulated LPS action in primary microglia and in microglia cell lines. Specifically, in agreement with our findings, opioids potentiated LPS-induced activation of NF-κB and the expression of cytokines in primary microglia (Merighi et al., 2013; Gessi et al., 2016), whereas they had the opposite effect in BV-2 cells or other microglia cell lines (Wang et al., 2018; Mali and Novotny, 2022; Shivling Mali et al., 2023). This discrepancy may reflect differences between rodent microglia cell lines and primary microglia that several studies have revealed, mainly following LPS stimulation. These studies have pointed out that, although microglia cell lines are suitable for biochemical and molecular approaches and for high-throughput screening assays which require high cell numbers, they differ both genetically and functionally from primary microglia (Butovsky et al., 2014; Das et al., 2016).

The innate immune receptor complex TLR4/MD-2 is a known sensor for LPS and other pathogen- and damage-associated molecular patterns. In addition, numerous studies on the opioid receptor-independent action of opioid ligands have shown that TLR4/MD-2 can respond to opioid drugs (Bettoni et al., 2008; Hutchinson et al., 2010; Wang X et al., 2012; Eidson and Murphy, 2013; Gabr et al., 2021). Binding of ligands causes dimerization of the TLR4/MD-2 extracellular domain and the subsequent recruitment of specific adaptor proteins to the intracellular domain, thus initiating a signaling cascade (Kawasaki and Kawai, 2014). Blocking the binding of TLR4/MD-2 ligands to the receptor or interfering with its intracellular signaling are the two major strategies to achieve the inhibition of TLR4/MD-2 complex (Gao et al., 2017). In this context, we have recently shown that PEA-OXA, the oxazoline derivative of palmitoylethanolamide, can accommodate into the binding pocket of MD-2 occupying a relevant portion of the LPS binding site and resulting in the suppression of LPS-induced proinflammatory signaling (Facci et al., 2023). Based on these findings, to explore whether fentanyl may target TLR4/MD-2, here we inhibited the microglia receptor complex with PEA-OXA. The compound reduced IL-1β production and secretion from microglia co-treated with LPS and fentanyl, suggesting the engagement of TLR4/MD-2 complex in the inflammatory effect of fentanyl. To further sustain the role of this receptor in fentanyl effects, microglia were also treated with CLI-095, a TLR4 specific inhibitor that binds to the amino acid Cys747 in the intracellular domain and disrupts the association with adaptor molecules, leading to the inhibition of signal transduction (Ii et al., 2006; Kawamoto et al., 2008; Takashima et al., 2009). CLI-095 blocked the production and release of IL-1β from microglia co-stimulated with LPS and fentanyl, confirming the role of TLR4/MD-2 in the modulation of fentanyl-induced microglia activation. Furthermore, given that CLI-095 completely blocked the inflammatory response of microglia, the effect of fentanyl appears exclusively mediated by the TLR4/MD-2 complex. This is of particular importance because, although the expression of GPCR opioid receptors in microglia is still controversial (Machelska and Celik, 2020; Missig et al., 2022), we have detected the expression of these receptors in primary microglia at the mRNA level. However, the addition of the high affinity opioid receptor antagonist naloxone (IC_50_ in the nanomolar range; Wang Q et al., 2012) did not modify microglia activation induced by LPS and fentanyl co-treatment. These data support the hypothesis that the proinflammatory effect of fentanyl is independent of its binding to GPCR opioid receptors.

To finally eliminate possible confounding effects caused by the presence of opioid receptors, we used HEK-Blue hTLR4 cells, that do not express these receptors and are usually used to examine drug effects targeted at TLR4/MD-2 complex (Stevens et al., 2013). When fentanyl was tested alone (*i.e*., in the absence of LPS), it did not change TLR4 activation; whereas co-treatment with LPS and fentanyl resulted in an increased activation of TLR4/MD-2 complex compared to LPS-induced receptor activation. Studies reporting the effects of opioid agents on TLR4 signaling using HEK-Blue hTLR4 cells have shown contradictory results: opioid receptor ligands *per se* do not modify or activate TLR4/MD-2 signaling, whereas when used with LPS, opioid ligands potentiate or inhibit LPS-induced receptor complex activation (Hutchinson et al., 2008; Stevens et al., 2013; Skolnick et al., 2014; Gabr et al., 2021). Here we confirm some, but not all the mentioned studies. Differences in opioid ligands, concentrations, and incubation times used in different laboratories might account for the discrepancies in the findings.

Macrophages are the peripheral immune cell type that more closely shares functional roles and immune signaling with microglia (Bachtell et al., 2015). Opioids affect a variety of macrophage functions, such as chemotaxis, phagocytosis, and cytokine synthesis and secretion (van Epps and Saland, 1984; Machelska and Celik, 2020). In addition, inflammatory cytokines and exogenous opioids stimulate mononuclear immune cells to release opioid peptides, which in turn recruit blood monocytes into the site of injury (Kuis et al., 1991; Plein and Rittner, 2018), suggesting that a reciprocal interaction occurs between immune cells and opioids. Previous studies have shown that opioids, either directly *via* opioid receptors or indirectly modulating the LPS-TLR4/MD-2 signaling, affect the immunoinflammatory response in macrophages and other innate immune cells (Wen et al., 2022; Sun et al., 2023). Here we reported that fentanyl treatment significantly increased LPS-induced expression and release of inflammatory cytokines from human MDM expressing barely detectable mRNA levels of MOP, DOP, and KOP receptors. Notably, we also showed that fentanyl in combination with LPS promoted rapid enhancement of NF-κB signaling, supporting the view that fentanyl and LPS activate common proinflammatory signaling downstream of TLR4/MD-2. It is possible to hypothesize that longer exposure to LPS and/or higher concentrations of distinct opioids than those tested here would lead to a hyporesponsive phenotype, which may partially account for the contradictory findings reported in previous studies (Delgado-Vélez et al., 2008; Izzi et al., 2008).

Collectively, we have shown the role of TLR4/MD-2 complex as the target for fentanyl-induced inflammatory responses. In the CNS, the inflammatory events are associated with alterations of neuronal homeostasis, particularly involving the glutamate neurotransmission (Haroon et al., 2017), which is of central importance to CNS activity, pain transmission, and opioid analgesic efficacy (Hutchinson et al., 2011). Accordingly, the optimization of small-molecule inhibitors of TLR4/MD-2-mediated immunocompetent cell activation may provide novel strategies for future development of effective and safe pharmacological agents for pain management. Pharmacological intervention using TLR4/MD-2 inhibitors has been a challenging approach for the last few decades. Despite their efficacy in preclinical studies, the candidates failed in different stages of clinical trials (Anwar et al., 2019). Previously, we showed that PEA-OXA has anti-neuroinflammatory effects that involve its activity against TLR4/MD-2 signaling (Facci et al., 2023). Here we further demonstrated that PEA-OXA is effective in blocking fentanyl-induced microglia activation and TLR4/MD-2 signaling, suggesting a potential of this molecule as adjunct to opioid therapy, alleviating adverse effects or potentiating the drug efficacy. We are aware of some limitations of this study, such as the use of *in vitro* approaches that do not recapitulate the *in vivo* biotransformation processes and the complex cell interactions occurring in chronic pain. However, the results obtained in this study might lay the ground for future assessment of suitable molecules in animal models of chronic pain.

## Data Availability

The original contributions presented in the study are included in the article/Supplementary Material, further inquiries can be directed to the corresponding author.

## Abbreviations

CD14, Cluster of differentiation 14; CI, Confidence intervals; DMEM, Dulbecco’s modified eagle medium; DMSO, dimethyl sulfoxide; DOP, δ opioid receptor; FBS, Fetal bovine serum; GPCRs, G protein-coupled receptors; HEK, Human embryonic kidney; IL, Interleukin; iNOS, Inducible nitric oxide synthase; KOP, κ opioid receptor; LPS, Lipopolysaccharide; MCSF, Macrophage colony stimulating factor; MD-2, Myeloid differentiation factor 2; MDM, Monocyte-derived macrophages; MOP, µ opioid receptor; NO, Nitric oxide; NOP, Nociception/orfanin FQ opioid peptide receptor; OD, Optical density; PCR, Polymerase chain reaction; PEA-OXA, *N*-Palmitoylethanolamine-oxazoline; SEAP, Secreted embryonic alkaline phosphatase; SEM, Standard error of the mean; TLR4, Toll-like receptor 4; TNF, Tumor necrosis factor.
