# Supplementary material for: Fentanyl enhances immune cell response through TLR4/MD-2 complex
Source: Front Pharmacol. 2024 Oct 9;15:1468644. doi: 10.3389/fphar.2024.1468644 (PMC11496304; doi:10.3389/fphar.2024.1468644)
Supplement: Supplementary file 1 [file DataSheet1.docx]

Supplementary Material

**1 Material and methods**

**1.1 Cell viability assay**

Microglia were seeded in poly-L-lysine coated 96-well plates in growth medium and allowed to adhere overnight. Growth medium was replaced with serum-free medium and cells were exposed to increasing concentrations of fentanyl (1-100 μM) for 6 and 24 h. After the incubation period, the medium was removed and the cells were incubated with MTT (0.18 mg/mL) in a humidified incubator at 37°C for 1 h. Afterwards, the supernatants were removed and the formazan crystals developed in the viable cells were solubilized with DMSO. The plates were then read on a microplate reader (Victor2 Multilabel Counter, Wallac, Cambridge, MA, USA) using a test wavelength of 570 nm and a reference wavelength of 630 nm.

**2 Figures**

**
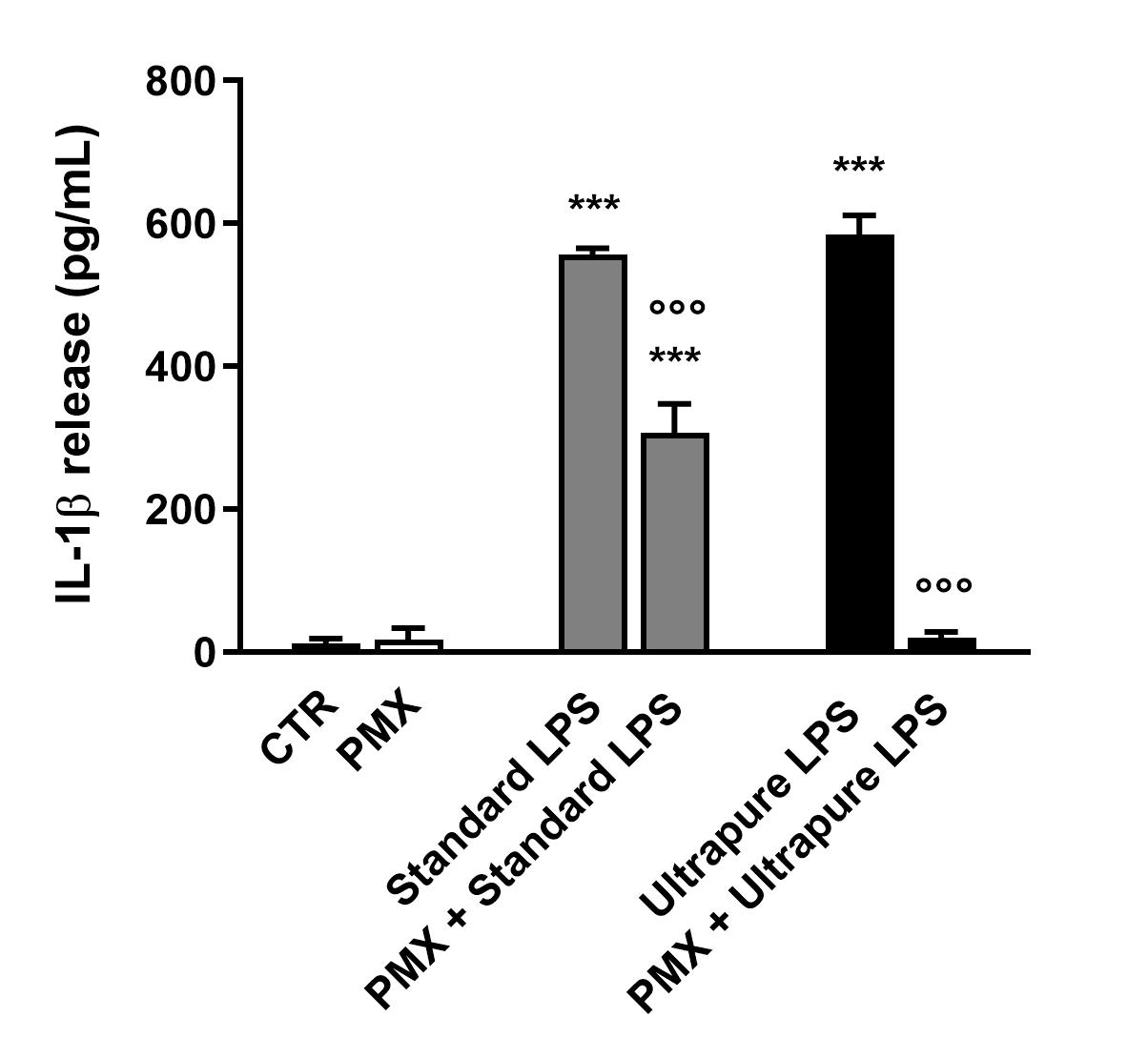
**

**Figure S1.** Effect of polymyxin B on IL-1β release from primary microglia. Microglia were cultured in 10% serum-containing medium, which was replaced with serum-free medium before treatment with 1 µg/mL of a standard preparation of LPS (gray bars) or ultrapure LPS (black bars) ± polymyxin B (PMX, 10 µg/mL) for 24 h. Supernatants were collected and analyzed for IL-1β release. Results are shown as means ± SEM (n = 3) and analyzed by one-way ANOVA followed by Holm-Sidak’s multiple comparison test. ***p < 0.001 compared to control cells (CTR); °°°p < 0.001 *vs* LPS stimulation.


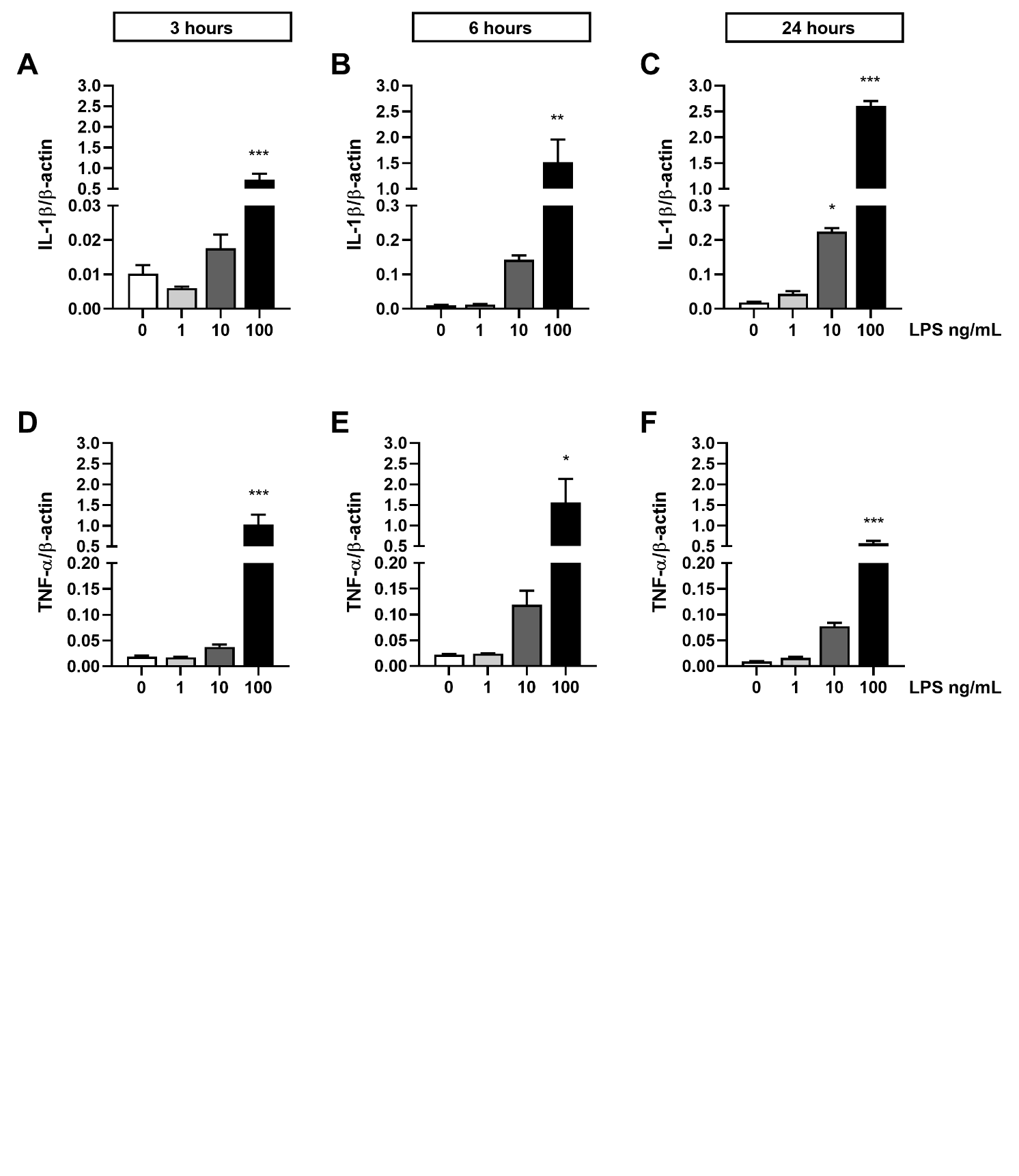


**Figure S2.** Time-course and concentration-response of LPS-induced gene expression of proinflammatory cytokines in primary microglia. Cells were cultured in 10% serum-containing medium, which was replaced with serum-free medium before treatment with increasing concentrations of LPS (1-100 ng/mL) for 3, 6, or 24 h. (**A-C**) IL-1β and (**D-F**) TNF-α mRNA levels were quantified by real-time PCR. Data are presented as means ± SEM (n = 3) and analyzed by one-way ANOVA followed by Holm-Sidak’s multiple comparison test. *p < 0.05, **p < 0.01, and ***p < 0.001 compared to control cells (white bars).


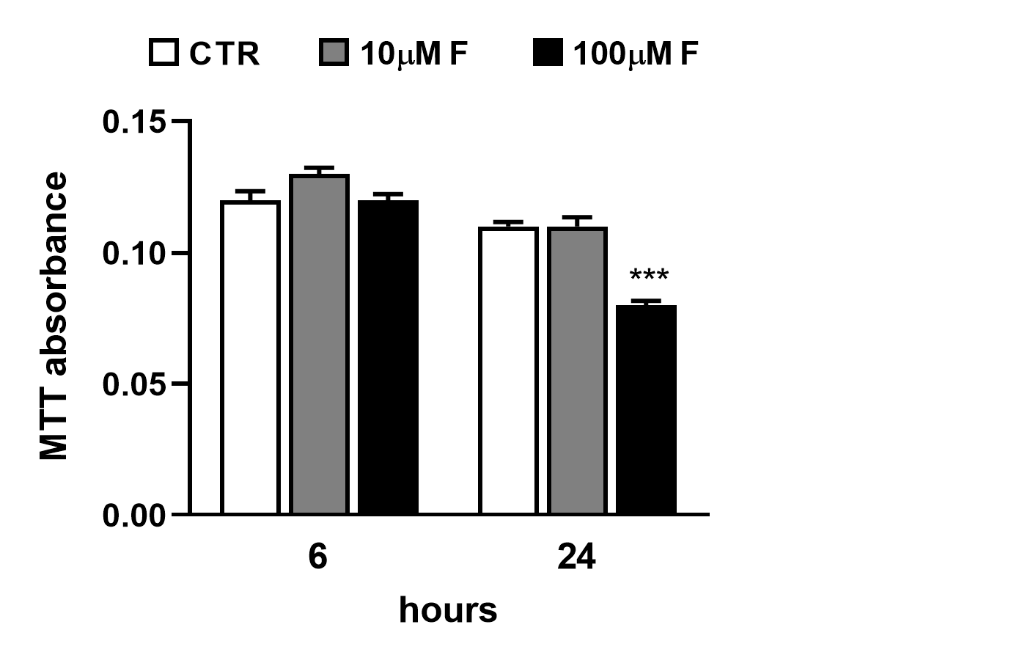


**Figure S3.** Effect of fentanyl on microglia cell viability. Microglia were cultured in 10% serum-containing medium, which was replaced with serum-free medium before treatment with increasing concentrations of fentanyl (1-100 µM) for 6 or 24 h. At the end of incubation, cell viability was determined by the MTT assay. Data are shown as means ± SEM (n = 3) and analyzed by two-way ANOVA followed by Dunnett’s multiple comparison test. ***p < 0.001 *versus* the corresponding control (white bar).
